# Effect of the GO Reduction Method on the Dielectric Properties, Electrical Conductivity and Crystalline Behavior of PEO/rGO Nanocomposites

**DOI:** 10.3390/polym9110613

**Published:** 2017-11-14

**Authors:** Asish Malas, Avanish Bharati, Olivier Verkinderen, Bart Goderis, Paula Moldenaers, Ruth Cardinaels

**Affiliations:** 1 Soft Matter Rheology and Technology, Department of Chemical Engineering, KU Leuven, Celestijnenlaan 200F, Box 2424, B-3001 Leuven, Belgium; malasasish@gmail.com (A.M.); avanishbharati413@gmail.com (A.B.); 2 Chemistry and Materials, Department of Chemistry, KU Leuven, Celestijnenlaan 200F, Box 2404, B-3001 Leuven, Belgium; olivier.verkinderen@kuleuven.be (O.V.); bart.goderis@kuleuven.be (B.G.); 3 Polymer Technology, Department of Mechanical Engineering, Eindhoven University of Technology, Box 513, 5600MB Eindhoven, The Netherlands

**Keywords:** reduced graphene oxide (rGO), polyethylene oxide (PEO), rheology, electrical conductivity, dielectric properties, crystallization

## Abstract

The effect of the reduction method to prepare reduced graphene oxide (rGO) on the melt linear viscoelastic properties, electrical conductivity, polymer matrix crystalline behavior and dielectric properties of PEO-rGO nanocomposites was investigated. Reduction was performed chemically with either sodium borohydride (NaBH_4_) or hydrazine monohydrate (N_2_H_4_·H_2_O) or both reduction agents consecutively as well as thermally at 1000 °C. The different reduction methods resulted in exfoliated rGO sheets with different types and amounts of remaining functional groups, as indicated by FT-IR, Raman, TGA and XRD characterization. Moreover, their electrical conductivity ranged between 10^−4^ and 10^−1^ S/cm, with the consecutive use of both chemical reduction agents being far superior. PEO nanocomposites with filler loadings of 0.5 wt %, 1 wt % and 2 wt % were prepared by solvent mixing. The rGO fillers affected the melt linear viscoelastic and crystalline behavior of the PEO matrix and resulted in nanocomposites with a substantially increased electrical conductivity. Despite the wide variability in filler conductivity, the effects on the polymer nanocomposite properties were less distinctive. A correlation was obtained between the reduction of the mobility of the polymer chains (evaluated by the glass transition temperature) and the dielectric strength of the interfacial polarisation originating from the effective entrapment of GO/rGO filler charges at the interface with the less conductive PEO. Thus, favorable interactions of the polar PEO with the filler led to reduced mobility of the PEO chains and thereby a more effective entrapment of the filler charges at the PEO interface.

## 1. Introduction

Conducting polymer nanocomposites have been extensively used as materials for electromagnetic interference (EMI) as well as radio frequency interference (RFI) shielding in electronic devices, for electrostatic dissipation, in chemical sensors, as anti-static materials, fuel cells, etc. [1,2]. However, inclusion of considerable amounts of filler particles in a polymer matrix can result in inferior mechanical performance and low processability. Therefore, a low electrical percolation threshold is advantageous to balance the various properties. The spatial distribution and shape of the filler particles are crucial to achieve a very low percolation threshold. Nanometric fillers with anisotropic shape are known to be very effective in this respect [3]. Graphene, a monolayer of sp^2^-hybridized carbon atoms arranged in a two-dimensional framework, has attracted huge attention because of its outstanding mechanical, thermal and electrical properties [3,4,5]. The electrical percolation threshold for graphene-filled polymer composites is generally low due to the large surface/volume ratio [3]. However, graphene flakes are much likely to restack with each other due to the van der Waals force of attraction. Hence, it is very difficult to obtain uniformly dispersed graphene sheets within a polymer matrix. Natural graphite powder is the starting material for the synthesis of graphene. Graphite powder is usually treated with a number of oxidizing agents in aqueous medium to exfoliate the graphene sheets of graphite [6]. Due to the vigorous oxidation, several functional groups (–COOH, –OH, epoxide, –CHO groups, etc.) are attached on the edges and in the backbone of the graphene sheets, which disrupt the conjugation as well as electronic mobility throughout the plane. Hence, to restore the electrical conductivity in GO, the reduction of functional groups is required. Several reduction methods, like chemical reduction [7], thermal annealing under inert atmosphere [8], and UV irradiation [9], are employed to reduce GO and prepare rGO. Since GO sheets are not prone to restacking with each other, in situ reduction of the dispersed GO flakes inside the polymer matrix can be a useful method to avoid restacking [10]. Thomassin et al. [11] synthesized GO/PMMA nanocomposites via in situ precipitation polymerization in the presence of GO. They showed that in situ thermal reduction for 1 h at 210 °C in a rheo-dielectric setup was almost as effective as a 5 min chemical reduction with hydrazine.

Nanocomposites of polyethylene oxide (PEO) with GO or rGO are suitable for a wide range of applications. PEO/rGO nanocomposites prepared by in situ chemical reduction in the PEO/rGO solution were shown to exhibit excellent microwave absorption properties [12]. Furthermore, by replacing the chemical reduction agent hydrazine with l-ascorbic acid, a green approach was developed to prepare PEO/rGO nanocomposites for EMI shielding [13]. Besides EMI shielding, the applicability of PEO/rGO and PEO/GO nanocomposites for toluene sensing [14], polymer electrolyte fuel cells [15] and flexible thin-film batteries [16] was investigated. Also shape-memory behavior could be introduced by preparing PEO/GO nanocomposites [17]. Mechanical properties of PEO can be tuned by using rGO in combination with an organic salt [18] and it was shown that graphene can nucleate PEO crystallization and affect the d-spacing of the PEO crystallites [19].

In all these studies, only one specific reduction procedure for rGO was used. However, recently Alazmi et al. [20] have shown that using different reduction methods for rGO such as thermal reduction, chemical reduction with hydrazine and hydrothermal reduction can lead to rGOs with a wide variety of chemical fingerprints and structural characteristics. Subsequently, the performance of these different types of rGO as supercapacitor electrodes varied substantially [21]. Up to now, it is not known how the different characteristics of rGO fillers are reflected in the properties of polymer nanocomposites. The first aim of the present study is to systematically investigate the efficiency of various reduction methods to prepare electrically conductive rGO from GO. The second aim is to study the relations between the characteristics of the different rGO types and the melt rheology, crystalline behavior, dielectric properties and electrical conductivity of PEO nanocomposites containing rGO. Several chemical reduction methods (with either NaBH_4_, N_2_H_4_·H_2_O or both) as well as ex situ and in situ thermal reduction methods will be applied and the obtained rGOs will be characterized by means of Fourier transform infrared spectroscopy (FT-IR), Raman spectroscopy, X-ray photoelectron spectroscopy (XPS), X-ray diffraction (XRD) and conductivity measurements. Polymer nanocomposites consisting of a semi-crystalline PEO matrix filled with these rGO particles will be prepared by solution casting. Melt rheology, dielectric spectroscopy, differential scanning calorimetry (DSC), scanning transmission electron microscopy (STEM) and XRD characterizations will allow to analyze the microstructure and dispersion of the rGO particles in the polymer, the polymer chain mobility, particle-polymer dielectric interfacial relaxation, crystalline behavior and electrical conductivity of the nanocomposites. Correlations between on the one hand particle properties and particle–polymer interactions, and on the other hand bulk properties of the polymer nanocomposites, will be established.

## 2. Materials and Methods

### 2.1. Materials

Graphite powder with particle size <20 μm (CAS 7782-42-5), the starting material for the synthesis of GO, was procured from Sigma-Aldrich. Sulfuric acid (H_2_SO_4_) (CAS 7664-93-9, >95% pure) was purchased from Fisher Scientific. Phosphoric acid (H_3_PO_4_) (CAS 7664-38-2, ≥85 wt % in H_2_O), sodium borohydride (NaBH_4_) (CAS 16940-66-2, 99% pure), hydrazine monohydrate (N_2_H_4_·H_2_O) (CAS 7803-57-8, N_2_H_4_ 64–65%, 98% pure), ethanol (CAS 64-17-5, ≥99.8% pure) and chloroform (CAS 67-66-3, ≥99% pure) were bought from Sigma-Aldrich. Potassium permanganate (KMnO_4_) (CAS 7722-64-7, 98% extra pure) and hydrochloric acid (HCl) (CAS 7732-18-5, 37% solution in H_2_O) were obtained from Acros Organics. Hydrogen peroxide (H_2_O_2_) 35 wt % solution (CAS 7722-84-1) was provided by Chem-Lab NV. Poly (ethylene oxide) with average *M*_v_ = 600,000 (CAS 25322-68-3, 200–500 ppm BHT as inhibitor), the base polymer matrix for the preparation of the polymer nanocomposites, was obtained from Sigma-Aldrich (Overijse, Belgium).

### 2.2. Synthesis of Graphene Oxide (GO)

GO was synthesized by a modified Hummer’s method [22]. In short, graphite powder (3 g) was added to a 9:1 mixture of concentrated H_2_SO_4_/H_3_PO_4_ (360 mL:40 mL) and stirred for 1 h. Then the mixture was cooled to 4–5 °C and 6 weight equivalents of KMnO_4_ (18 g) were added to the mixture very slowly, producing a small exothermic response which increased the temperature to 35–40 °C. After that, the reaction mixture was stirred for 12 h at 50 °C and subsequently poured in a beaker containing 400 mL of ice water and 3 mL of H_2_O_2_ (35%). Then, the GO dispersion was centrifuged at 4000 rpm for 30 min and the supernatant liquid was decanted. The solid material was thoroughly washed with consecutively 400 mL deionized water, 200 mL 30% HCl solution, 400 mL deionized water, and 200 mL ethanol and centrifuged each time at 4000 rpm for 20 min. After that, the dark yellowish GO residue was dried in a vacuum oven at 60 °C for 24 h.

### 2.3. Synthesis of Reduced Graphene Oxide (rGO) by Different Reduction Methods

As a result of the vigorous oxidation of graphite, the double bonds in the graphene sheets lose their conjugated structure due to the incorporation of different functional groups. This is detrimental for the thermal and electrical conductivity of GO. To re-establish the conjugated structure of the graphene sheets in GO, the functional groups of GO were selectively reduced by using different reduction procedures.

In the first reduction procedure, GO was reduced with sodium borohydride (NaBH_4_), following the approach of Lee and Kim [23]. Yellowish brown GO powder was dispersed in deionized water (300 mg/300 mL) and bath-sonicated for 1 h in a round-bottom flask. After that, the GO dispersion was heated up to 90 °C in an oil bath. A NaBH_4_ solution (5 g/80 mL deionized water) was prepared freshly and added to the hot GO dispersion drop by drop. Then, the total solution was vigorously stirred for 4 h at 90 °C under a cold water circulating condenser, during which the solution color turned black. After that, the solution was cooled to room temperature, filtered (using filter paper with 5 μm particle retention ability) and washed with deionized water as well as washed with ethanol several times. In the end, the residue was dried in a vacuum oven for 24 h to obtain black shiny NaBH_4_ reduced GO (NarGO).

In the 2nd reduction method, GO was reduced with hydrazine monohydrate (N_2_H_4_·H_2_O), following the approach of Stankovich et al. [24]. GO (100 mg/100 mL) was dispersed in deionized water in a round-bottom flask and placed in an ultrasonic bath for 1 h. After that, the round-bottom flask containing the GO dispersion was transferred to an oil bath. N_2_H_4_·H_2_O (1 mL) was then poured into the GO dispersion and stirred. After that, the solution was heated to 100 °C under a water-cooled condenser for 24 h with constant stirring. The final black solution was cooled, filtered and washed several times with water and ethanol. Finally, the product was dried overnight under vacuum to obtain N_2_H_4_·H_2_O reduced GO (HyrGO).

To achieve maximum reduction of the functional groups of GO, in the 3rd reduction method, the wet residue of the 1st reduction process (NarGO synthesized from 300 mg GO) was transferred in another round-bottom flask containing 300 mL water. Subsequently, the procedure described for the 2nd reduction method was performed, using 3 mL N_2_H_4_·H_2_O. This resulted in GO reduced by two reducing agents (NaHyrGO). 

To compare the effect of the three chemical reductions with that of a thermal reduction of GO, 1 g of GO powder was transferred into a ceramic crucible and annealed at 1000 °C under inert atmosphere, similar to Song et al. [25]. The GO-containing ceramic crucible was heated from room temperature to 1000 °C at a rate of 5 °C/min and kept at 1000 °C for 6 h under an argon flow of 80 mL/min. After that, the crucible was naturally cooled to room temperature to obtain thermally reduced graphene oxide (TrGO).

### 2.4. Fabrication of Polymer Nanocomposites by Solution Mixing

Poly(ethylene oxide) (PEO)-based nanocomposites filled with graphite, GO and different rGOs (NarGO, HyrGO, NaHyrGO and TrGO) were prepared by solution casting at different filler loadings (0.5, 1 and 2 wt %). For the GO and chemically reduced GOs, the filler powder was primarily dispersed in deionized water by ultra-sonication for 1 h (GO) or 5 h (NarGO, HyrGO and NaHyrGO). This resulted in a uniform suspension without visible aggregates. In the meantime, PEO was dissolved in water at room temperature. After that, the dispersed filler solution was added to the polymer solution and the mixture was stirred for 3 h. The whole solution was then poured in a Petri dish and dried in a vacuum oven at 70 °C to obtain the nanocomposite film. Graphite and TrGO could not be dispersed in water. CHCl_3_ was used as solvent to disperse graphite and TrGO by 1 h ultra-sonication. Meanwhile, PEO was also dissolved in CHCl_3_. Both solutions were mixed together and stirred for 3 h. The overall solution was then poured in a Petri dish and dried in air to obtain the nanocomposite film. After that, the nanocomposite films were cut in pieces and subsequently compression-molded at 100 °C and 50 MPa for 8 min by using a laboratory scale plate press (Collin, Ebersberg, Germany), resulting in nanocomposite discs of 25 mm diameter and 1.5 mm thickness.

### 2.5. Characterization of the GO and rGO Fillers

To qualitatively determine the presence of different functional groups on GO and the extent of reduction of the various functional groups after different reduction methods (chemical/thermal), Fourier transform infrared spectra (FT-IR) of GO and different rGOs were recorded on a Bruker Vertex 70 spectrometer (Bruker Optics, Evere, Belgium). Powder samples were examined as such by utilizing a platinum ATR single-reflection diamond attenuated total reflection (ATR) component.

Raman spectroscopy was performed by using an inverted optical microscope (TiU, Nikon, Groot-Bijgaarden, Belgium) equipped with a piezoelectric stage (Physik Instrument (PI) GmbH & Co., Sint-Oedenrode, The Netherlands). Diffraction-limited focused excitation was provided by a continuous wave 632.8 nm He-Ne laser (Model 1145P, JDS Uniphase Co., San Jose, CA, USA). Laser light was guided to the sample through an appropriate dichroic mirror (z633rdc for 633 nm excitation, Chroma Technology Co., Olching, Germany). To obtain a diffraction-limited focus, collimated laser light was directed through the objective (×60, NA 0.98, PlanApo, Nikon, Tokyo, Japan). The Raman scattering from the sample was collected using the same objective and dichroic mirror. A long pass cutoff filter (optical density OD > 5 at the excitation wavelength) was employed before the detector to reject the excitation light (HQ645LP for 633 nm excitation, Chroma Technology Co., Olching, Germany). The Raman signals were recorded with a spectrometer (iHR 320, Horiba, Longjumeau CEDEX, France) equipped with a cooled electron multiplying charge-coupled device (CCD) camera (Newton 920, Andor, Belfast, UK).

To compare the thermal degradation behavior of GO and different rGOs, a thermogravimetric analyzer (TGA Q500, TA Instruments, Zellik, Belgium) was used to carry out thermal ramps under nitrogen atmosphere in the temperature range from room temperature to 500 °C at a heating rate of 10 °C/min.

X-ray diffraction patterns (XRD) of GO and rGOs were recorded at 25, 100 and 150 °C with a Xenocs Xeuss Mo (SWAXS) (Xenocs, Sassenage, France) device fixed with a Linkam DSC600 hotstage (Linkam Scientific Instruments, Tadworth, UK). Tzero aluminum pans with a hermetic lid were used as sample holder. The setup uses a Molybdenum source with a typical Kα wavelength of 0.7117 Å and was operated at 50 kV and 1 mA. The d-spacings of graphite, GO and rGO were determined using Bragg’s equation:

(1) nλ=2dsinθ

where *n* is an integer (order of diffraction), *λ* is the wavelength, *d* is the interlayer spacing and θ is the scattering angle.

The electrical conductivity of GO and the different rGOs was determined at 100 and 150 °C with a rheo-dielectric setup consisting of a dielectric analyzer (Novocontrol Technologies, Montabaur, Germany) combined with an MCR501 stress controlled rheometer (Anton Paar, Graz, Austria) with a CTD450 temperature controlled convection oven under nitrogen atmosphere. The plates of the rheometer acted as electrodes for dielectric experiments. The powder samples were compression-molded into discs (8 mm diameter and 1 mm thickness) at room temperature and 50 MPa. Conductivity spectroscopy measurements were carried out in the frequency range of 10^−2^ to 10^7^ Hz. The results were evaluated by extracting the complex conductivity from the complex impedance values [26,27].

### 2.6. Characterization of the Nanocomposites

The melt state linear viscoelastic properties of the different GO-filled PEO nanocomposites were investigated with an AR2000 stress-controlled rheometer (TA Instruments, Zellik, Belgium). A parallel plate geometry (25 mm diameter and 1.5 mm sample thickness) was used for small amplitude oscillatory shear measurements under N_2_ atmosphere at 150 °C. Initially, an oscillatory time sweep experiment at a fixed angular frequency (ω) of 1 rad/s and 1% strain (within the linear viscoelastic regime) was performed to determine the time required to obtain steady-state moduli. An initial small increase in the moduli resulted from network development during annealing at elevated temperature. After a constant value of the storage modulus (*G*–) was attained, a small amplitude oscillatory frequency sweep experiment was performed in the range of 0.01 to 100 rad/s at 1% strain and 150 °C.

The dispersion of various rGOs in the polymer matrix and the effect of the different fillers on the crystalline behavior of semi-crystalline PEO were determined by XRD on compression-molded nanocomposites, using the same setup and sample holder as for the fillers. The percentage of crystallinity χ_c,XRD_ (on volume basis) of the different PEO-based nanocomposites was determined as:

χ_c,XRD_ = [*I*_c_/(*I*_a_ + *I*_c_)] × 100 (2)

where *I*_a_ and *I*_c_ are the integrated intensity of the amorphous and crystalline region respectively. Images were corrected for dark current and background and scaled to the same total integrated intensity.

For morphology analysis, ultrathin nanocomposite sections of 70 nm were sectioned below the glass transition temperature of PEO (−100 °C) by using a diamond knife mounted in an ultra-microtome (LEICA ULTRACUT UCT, Diegem, Belgium) under cryogenic conditions. After that, the sample sections were mounted on a carbon-coated copper grid (150 mesh). Imaging was done using a scanning electron microscope (SEM, FEI Nova NanoSEM 450, Hillsboro, OR, USA) operated in STEM mode, which provides better contrast between the matrix and filler as compared to normal SEM mode. The images were taken in bright field transmission mode at an accelerating voltage of 20 kV.

To study the effect of GO and rGO on the crystalline behavior of PEO, differential scanning calorimetry (DSC) was performed for the different nanocomposites in a Q2000 DSC (TA Instruments, Zellik, Belgium). Samples (~10 mg) were dried in a vacuum oven before the experiments. The samples were initially heated from room temperature to 110 °C (heating rate 10 °C/min) and were then held for 10 min at this temperature to eliminate previous thermal history. After that, the samples were cooled to room temperature and again reheated (2nd scan) to 110 °C at the same rate. The melting temperature (*T*_m_), heat of fusion (∆*H*_m_) and non-isothermal crystallization temperature (*T*_c_) of neat PEO and PEO-based nanocomposites were determined. The degree of crystallinity χ_c,DSC_ (on mass basis) of the different nanocomposites was calculated from:

χ_c,DSC_ = 100 × (∆*H*_m_/∆*H*^0^_m_)/w (3)

where ∆*H*^0^_m_ is the heat of fusion of 100% crystalline PEO (205 J/g) [28,29] and w is the weight fraction of PEO in the nanocomposites. To determine the *T*_g_ of PEO and the nanocomposites with various fillers, heating scans starting from −80 °C were performed, in which the *T*_g_ was determined from the peak of the derivative of the heat capacity.

In order to show the structural relaxations of PEO and the dielectric interfacial relaxations originating from the (r)GO—PEO interface in the nanocomposites, dielectric spectroscopy was performed with the rheo-dielectric setup on disks with a diameter of 8 mm and thickness of 1 mm. Both the complex conductivity and the complex permittivity were evaluated from the complex impedance values [26,27]. The contribution of the conductivity to the dielectric loss spectra was eliminated by calculating the dielectric loss from the dielectric constant using the logarithmic derivative technique [27].

## 3. Results and Discussion

### 3.1. Analysis of FT-IR Spectra of Graphite, GO and Different rGOs

**Figure 1:**
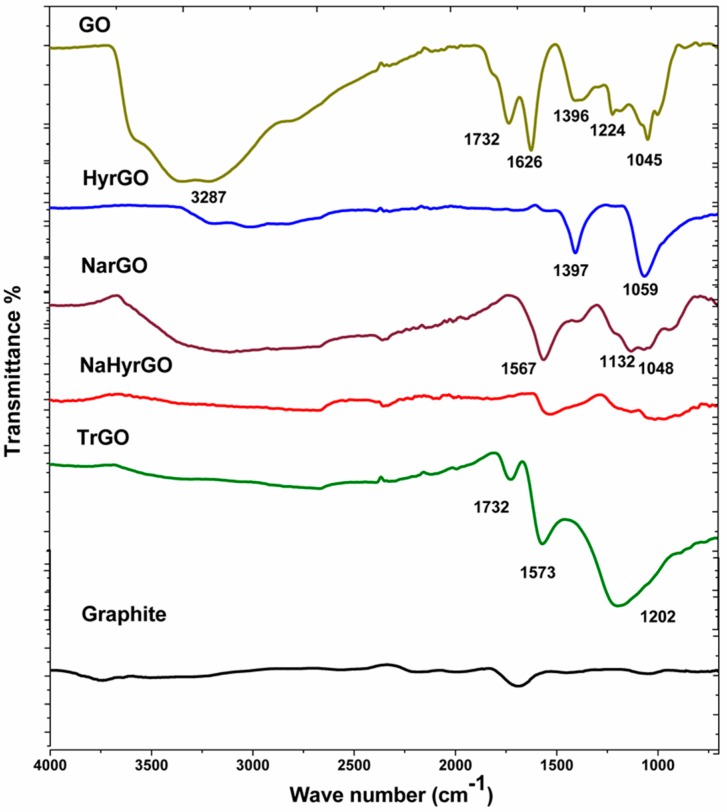
FT-IR spectra of graphite, GO and different reduced GOs.

To determine the presence of different functional groups on the surface as well as in the backbone of GO (due to the vigorous oxidation) and to qualitatively investigate the reduction of the functional groups of GO by the application of different chemical and thermal reduction methods, FT-IR spectra were collected. The FT-IR spectra of the different fillers are shown in Figure 1. For GO, the absorption peaks can be attributed to –OH stretching vibrations (from intercalated water molecules as well as hydroxyl groups) (broad band around 3287 cm^−1^), –C=O stretching vibrations (1732 cm^−1^), aromatic C=C vibrations (1626 cm^−1^), O–H bending vibrations of tertiary C–OH (1396 cm^−1^) as well as C–O stretching vibrations (1224 cm^−1^ and 1045 cm^−1^) [20,30,31,32]. The FTIR spectrum of the graphite used as the starting material for the preparation of GO does not show the presence of any oxygen-containing functional groups. For the hydrazine-reduced GO (HyrGO), the broad band around 3200 cm^−1^ corresponding to the stretching vibrations of –OH is strongly reduced whereas no peaks remain related to the C=O bonds. The –OH bending vibration of tertiary C–OH (1397 cm^−1^) as well as the C–O stretching vibration (1059 cm^−1^) remain present. For NaBH_4_ reduced GO (NarGO), the broad band around 3200 cm^−1^ corresponding to stretching vibrations of –OH is slightly more pronounced as for the HyrGO and also the aromatic C=C vibrations (1567 cm^−1^) and C–O stretching vibrations (1132 and 1048 cm^−1^) can still be discerned. For the NaBH_4_/hydrazine reduced GO (NaHyrGO), it can be seen from the spectrum that the peaks corresponding to the different functional groups of GO are no longer present, which points to an efficient reduction of the various functional moieties of GO by the dual reducing agents (NaBH_4_/N_2_H_4_·H_2_O). The FT-IR spectrum of TrGO exhibits a substantially diminished peak corresponding to –OH groups. In addition, the presence of C=O bonds (1732 cm^−1^), aromatic C=C bonds (1573 cm^−1^) and C–O bonds (1202 cm^−1^) is clear. Similarly, Hsiao et al. [33] observed the presence of –OH, –COOH and epoxy groups in TrGO synthesized by thermal reduction at 1050 °C under inert atmosphere.

### 3.2. Analysis of Raman Spectra of Reduced GOs Synthesized by Different Methods

**Figure 2:**
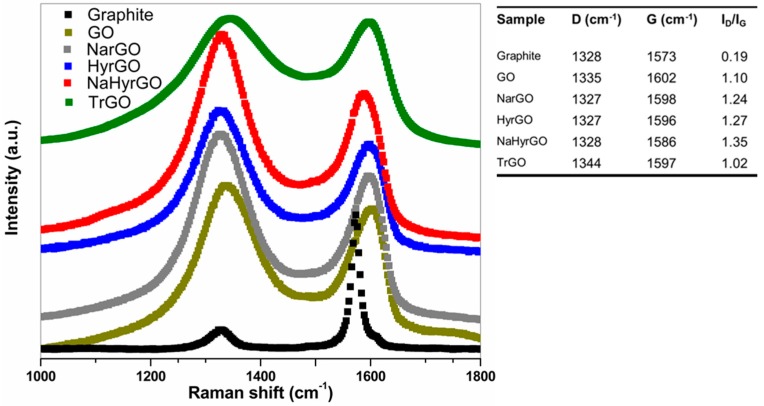
Raman spectra of graphite, GO and different reduced GOs.

Raman spectroscopy is an effective tool to analyze the relative contributions of ordered and disordered regions in carbonaceous structures, thereby allowing us to comment on the skeletal changes of the GO and different rGOs during oxidation and subsequent reduction step(s). Figure 2 shows the Raman spectra of graphite, GO and different rGOs. The graphite lattice exhibits a characteristic G band at 1573 cm^−1^, which is generally assigned to C=C stretching vibrations common to all sp^2^ bonded carbon atoms, whereas a weak D band at 1328 cm^−1^ originates from phonon modes coming from sp^2^ bonded carbon atoms that reside near local lattice distortions (defects) of the graphitic network [34,35]. Hence, the intensity ratio of the D band and the G band (*I*_D_/*I*_G_) allows to evaluate the transformations between ordered and disordered structures [34]. Due to the vigorous oxidation of GO, a pronounced D band occurs at 1335 cm^−1^, which reflects the reduction in size of the ordered in-plane sp^2^ domains due to the attachment of functional groups. Simultaneously, the G band is widened and moved to 1602 cm^−1^, which could originate from several factors but the most plausible one is the generation of isolated double bonds on GO flakes that vibrate at higher frequencies as compared to the conjugated double bonds of graphite sheets [35].

After the reduction of GO by different chemical procedures, the G band moves back close to its initial position in graphite, suggesting the reappearance of the graphitic sp^2^-conjugated structure. On the other hand, the *I*_D_/*I*_G_ ratios for all the chemically reduced GO samples are larger than that of GO. This implies a reduction in the domain size of sp^2^ bonded carbon atoms in the various rGO flakes, which may appear counter-intuitive. However, this increase of *I*_D_/*I*_G_ with increasing extent of reduction was also observed by Zhang et al. [36] for increasing treatment times with hydrazine hydrate and by Marciano et al. [37] for increasing reduction temperatures during thermal reduction. The latter authors suggest that the removal of oxygen groups can cause dangling bonds that introduce sp^3^ carbons. Another possibility is that reduction increases the overall presence of regions of sp^2^ bonded carbon atoms but reduces their size [38]. XPS spectra were collected to shed more light on the hybridization state of carbon. However, the amount of sp^2^ and sp^3^ carbons could not be determined separately due to too close overlap of the peaks (see Supplementary Information and Figure S1). NaHyrGO exhibits the highest *I*_D_/*I*_G_ value and a maximum shift towards the graphitic character (G band shift towards lower frequency) compared to the other reduced GOs. This confirms the conclusion from the FTIR analysis that combining various reducing agents is the most effective reduction method. Contrary to the chemically reduced rGOs, the *I*_D_/*I*_G_ ratio for TrGO is even smaller than that of GO. It is accepted that a Raman spectrum of reduced GO with smaller *I_D_*/*I*_G_ ratio compared to GO signifies repair of defects resulting in a larger distance between them [39]. Despite the larger sp^2^-domain size, the presence of non-negligible amounts of –OH and –COOH groups in TrGO (Figure 1) makes it less graphitic in nature, which is also observed from its G band shift in Figure 2.

### 3.3. Thermal Degradation Behavior of Graphite, GO and rGOs

**Figure 3:**
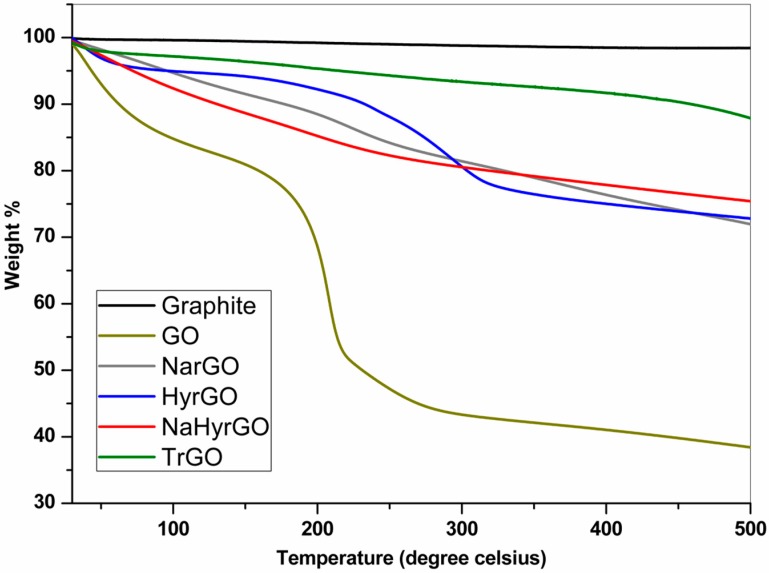
TGA curves of graphite, GO and different reduced GOs.

The thermal degradation behavior of GO, TrGO, NarGO, HyrGO and NaHyrGO was investigated by TGA, as shown in Figure 3. The weight loss of GO up to 100 °C can be attributed to the elimination of water molecules captured inside the hydrophilic GO framework. The main mass loss at around 200 °C can be assigned to the thermal decomposition of labile oxygen-containing groups of GO in the form of different gases (CO, CO_2_ and steam) [40]. The different chemically reduced rGOs display a much higher amount of residue as compared to GO due to the fact that a certain amount of the different functional groups was already removed by the chemical reduction. All rGOs show a substantial reduction of the main mass loss at 200 °C, with in the case of HyrGO predominantly the mass loss in the temperature range of 250 °C to 300 °C remaining, whereas a limited gradual mass loss over the whole temperature range remains for NarGO and NaHyrGO. Although the FT-IR and Raman spectroscopy analyses identified clear differences in the type of functional groups remaining in the rGOs prepared by different chemical reduction methods, the nearly equal final mass of these rGOs indicates a very similar total level of remaining functional groups. TrGO, on the other hand, shows a higher thermal stability as compared to the chemically reduced rGOs, which is due to the removal of a maximum amount of functional groups by thermal reduction at 1000 °C except for some –COOH groups along with certain epoxide groups, as shown by the FT-IR results in Figure 1.

### 3.4. X-ray Diffraction Patterns of Graphite, GO and Various rGOs

**Figure 4:**
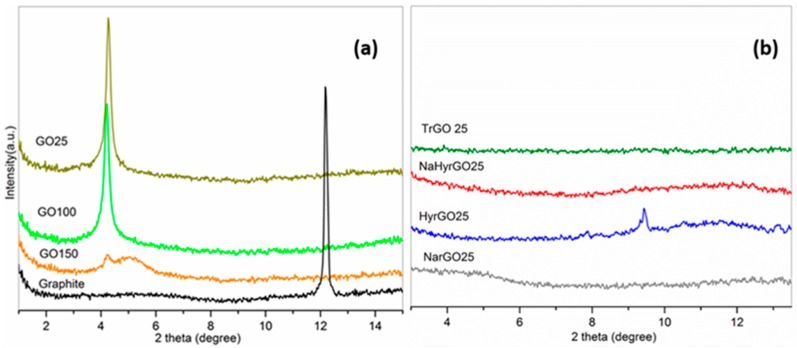
XRD patterns of (**a**) Graphite (at 25 °C) and GO at different temperatures (numbers indicate temperature), and (**b**) chemically and thermally reduced GOs at 25 °C.

To investigate the structural changes in graphite due to the vigorous oxidation and the subsequent reduction of different functional groups by various chemical and thermal methods, XRD patterns at different temperatures are shown in Figure 4. The XRD pattern of graphite (Figure 4a) shows a peak at 2θ = 12.16° corresponding to a d-spacing (d_002_) of 0.335 nm, which corresponds to reported literature values [20,30]. After strong oxidation of graphite to GO, the (002) peak is shifted towards a lower angle 2θ = 4.26° corresponding to a d-spacing of 0.956 nm. The huge increase in the inter-gallery spacing confirms the presence of intercalated water molecules and different polar functional groups in GO. Interestingly, Figure 4a shows that at 150 °C the diffraction peak of GO is more or less diminished, which can be attributed to partial exfoliation due to the removal of entrapped water moieties and some oxygen functionalities from GO. Hence, partial reduction already occurs at this temperature, as was also noted by Thomassin et al. [11] for PMMA-GO nanocomposites heated to 210 °C. For the different chemically reduced rGOs and TrGO (Figure 4b) the intense GO peak at 2θ = 4.26° is absent, regardless of the temperature. This is caused by the exfoliation of the graphene sheets during the elimination of the intercalated water molecules and different oxygen-containing functional groups. For HyrGO partial restacking of the graphene layers, with a d-spacing of 0.433 nm (2θ = 9.42°), which is significantly lower than that of GO but a little higher than that of graphite, is observed.

### 3.5. Bulk Electrical Conductivity of Graphite, GO and rGO Powders at Different Temperatures

**Figure 5:**
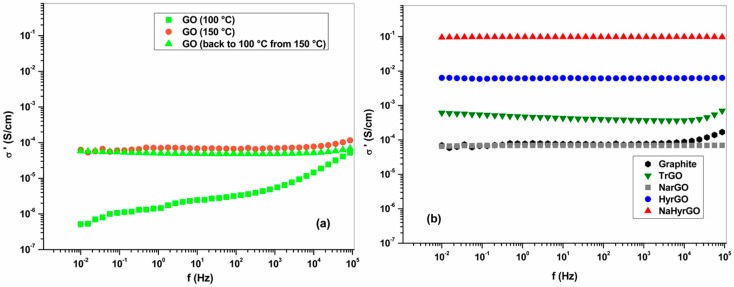
Bulk electrical conductivity of (**a**) GO at 100 and 150 °C, and (**b**) graphite and different rGOs at 150 °C.

The electrical conductivity of compressed pellets of the different fillers was measured at 100 °C and 150 °C. The electrical conductivity of GO, before and after heating to 150 °C, is shown in Figure 5a. Due to the presence of different functional groups on the surface and backbone of GO, it exhibits a moderate electrical conductivity at 100 °C. However, at 150 °C the electrical conductivity of GO is two decades higher, which can be attributed to the attainment of a partially conjugated structure due to the elimination of entrapped water molecules along with some of the labile functional groups at this temperature. The fact that this conductivity increase is effectively caused by irreversible structural changes is confirmed by the unaltered conductivity obtained after cooling the GO powder back to 100 °C. Figure 5b provides a comparison of the electrical conductivity of the different rGOs. The bulk electrical conductivity of different rGOs at 100 °C and 150 °C is comparable (data at 100 °C not shown). All powders show a nearly frequency-independent AC conductivity, which indicates the dominance of conduction over capacitive properties over a wide range of length scales [41]. Figure 5b shows that NaBH_4_ reduced GO (NarGO) shows an electrical conductivity of ~1 × 10^−4^ S/cm which is comparable to the electrical conductivity of GO at 150 °C. This indicates that the elimination of entrapped water molecules inside the GO lattices along with some functional groups by thermal reduction at 150 °C is comparable to the chemical reduction of different functional groups by NaBH_4_. The electrical conductivity of thermally reduced GO (TrGO) is ~1 × 10^−3^ S/cm which is ten times the conductivity of GO at 150 °C due to the extra removal of captured water molecules along with some of the functional groups at 1000 °C under inert atmosphere. The highest conductivity of ~0.1 S/cm is obtained for NaHyrGO which is due to the maximum elimination of functional groups (FT-IR spectra in Figure 1) as well as due to the attainment of a higher graphitic character (Raman spectra in Figure 2) as compared to the other reduced GOs. It should be noted that all exfoliated powders exhibit a conductivity that is equal to or larger than that of the graphite starting material (Figure 5b). This is in correspondence with the known larger single-particle conductivity of graphene versus graphite [42]. Nevertheless, at lower compression pressures as the one used in the present work, the contacts between particles may dominate, leading to lower conductivity values for compacted graphene powder as compared to compacted graphite powder [42].

### 3.6. Effect of GO and Different Reduced GOs on the Linear Viscoelastic Behavior of PEO

**Figure 6:**
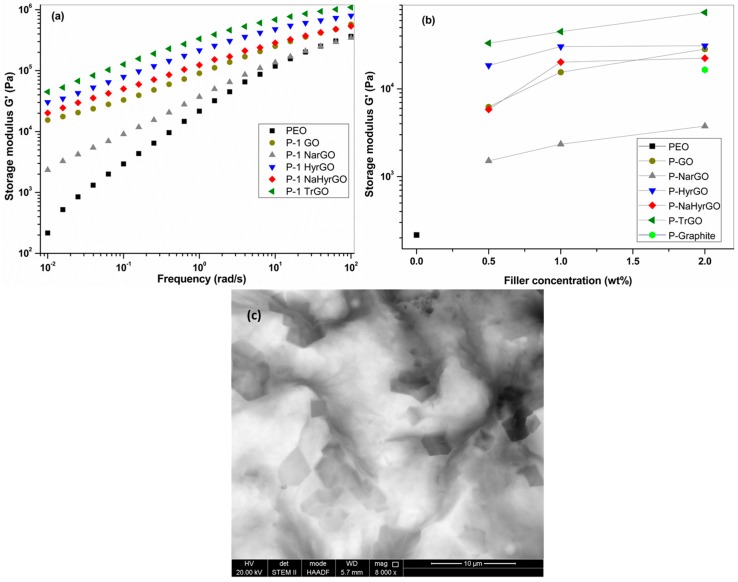
Viscoelastic properties (**a**) storage modulus (*G*–) at 1 wt % loading, (**b**) storage modulus (*G*–) at 0.01 rad/s versus filler concentration (wt %) of PEO-based nanocomposites with graphite, GO and rGO, and (**c**) representative STEM image of P-2 NaHyrGO nanocomposite.

Addition of nanofillers in a polymer matrix generally results in a significant increase in the melt viscoelastic moduli, possibly leading to a transition from liquid-like to solid-like behavior, the latter resulting in frequency-independent storage and loss moduli [3]. Therefore, rheology of polymer nanocomposites in the linear viscoelastic region allows to analyze the state of dispersion of the nanofiller in the polymer matrix [43]. The linear viscoelastic storage modulus of 1 wt % PEO-based GO and rGO nanocomposites at 150 °C and the dependence of the storage modulus on filler concentration (0.5, 1 and 2 wt %) are presented in Figure 6. It can be seen from Figure 6a that the storage modulus of neat PEO approaches a slope of 2 at low frequencies, indicating that terminal behavior is nearly reached [44]. PEO-based nanocomposites filled with GO and different rGOs show a substantial increase in the storage modulus (*G*–) value as well as a decrease of the low-frequency slope of the G– versus frequency curves, which indicates a reduced mobility due to interactions between the PEO chains and the GO/rGO sheets or partial network formation of the fillers [3]. Formation of fractal particle networks in polymer nanocomposites will result in a power law dependency of the storage modulus on the particle concentration [43]. Figure 6b shows that such behavior is not obtained for all different GO- and rGO-based nanocomposites. Even though the reduction of polar functional groups from GO could decrease the compatibility between polar PEO chains and the filler, all nanocomposites with rGOs show storage moduli of the same order of magnitude as that of the nanocomposite with GO. Only NarGO-filled PEO-based nanocomposites show less increase in storage modulus as well as more dependency of G– on frequency in the low frequency region indicating a poor dispersion of NarGO in PEO, i.e., the presence of an agglomerated structure. Figure 6b shows that for 2 wt % nanocomposites, the storage modulus of the nanocomposite with NarGO is even lower than that of a nanocomposite containing 2 wt % graphite. As a result of the thermal treatment of GO at 1000 °C, TrGO exhibits a more wrinkled or folded structure as compared to chemically reduced rGOs, which may explain its improved dispersion. On the other hand, it should also be kept in mind that this nanocomposite was prepared with another solvent (CHCl_3_) due to its reduced polarity. A representative STEM image (for the PEO nanocomposite with NaHyrGO sheets) is shown in Figure 6c. No large aggregates can be observed in this image, indicating a relatively uniform dispersion of the NaHyrGO. Between the different filler types, no differences could be discerned from the STEM images, confirming that rheology is a more sensitive tool to characterize filler dispersion.

### 3.7. Effect of GO and Different rGOs on the Crystalline Behavior of PEO

**Figure 7:**
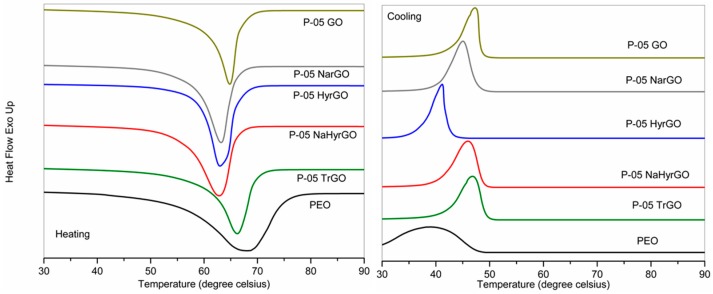
DSC thermograms of 0.5 wt % PEO-based nanocomposites.

**Figure 8:**
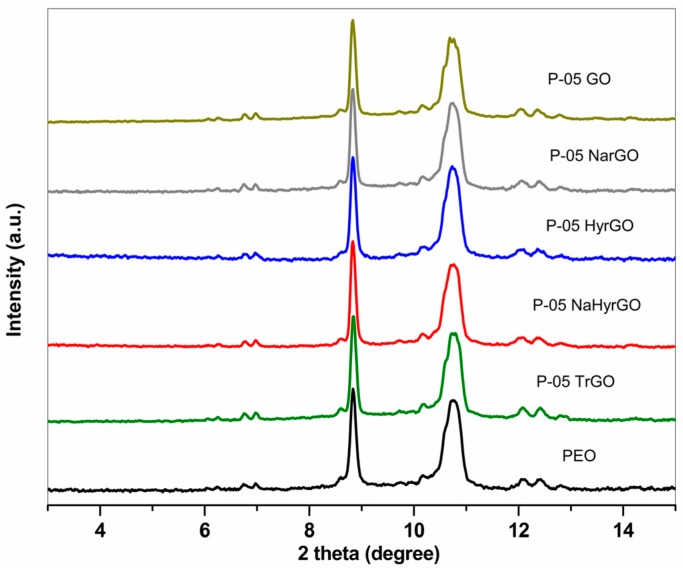
XRD patterns of 0.5 wt % PEO-based nanocomposites at 25 °C.

Differential scanning calorimetry (DSC) experiments were carried out for the PEO-based nanocomposites to observe the effect of the different rGOs on the crystalline behavior of PEO. The DSC thermograms of the different 0.5 wt % filler loaded nanocomposites are shown in Figure 7. The melting temperature (*T*_m_), crystallization temperature (*T*_c_), onset crystallization temperature (*T*_onset_ _cryst._), heat of melting (∆*H*_m_) and degree of crystallinity (χ_c,DSC_) acquired from the DSC graphs of the polymer nanocomposites (for 0.5 and 2 wt % loadings) are presented in Table 1. The degree of crystallinity of PEO filled with different fillers was also determined by analyzing the XRD patterns. The XRD patterns of 0.5 wt % GO or rGO loaded PEO-based nanocomposites are shown in Figure 8 and clearly exhibit the reflections of the 112 and 032 (2θ = 8.82°) as well as the 120 (2θ = 10.76°) crystallographic planes of PEO [45]. The calculated degrees of crystallinity of the different nanocomposites based on these peaks are tabulated in Table 1.

Table 1 clearly shows that the crystallization temperature of PEO is increased due to the addition of the GO or rGOs. This indicates that the fillers act as nucleation sites for crystallization [19]. In addition, the degree of crystallinity of PEO decreased due to the incorporation of different rGOs. This is caused by the fact that the molecular mobility of the polymer chains is reduced by the GO and rGO which also form barriers that restrict crystallization [17,19,46]. Unexpectedly, the nanocomposite with 0.5 wt % HyrGO shows a lower crystallization temperature than pure PEO. Such an anti-nucleation effect has been reported in literature for different types of nanocomposites such as cellulose nanocrystals in poly(oxyethylene) [28] or poly(epsilon)caprolactone [47] or functionalized multiwall carbon nanotubes in poly(epsilon)caprolactone [48]. This effect was attributed to reduced polymer chain mobility [28] or reduced encounters between polymer chains due to dilution [47]. However, Table 2 also shows that the degree of crystallinity for this nanocomposite was not reduced, which is generally the case when molecular mobility is suppressed. It should be noted though that due to the relatively large cooling and heating rate of the DSC experiments, kinetic effects may also play a role since fillers not only affect the crystal nucleation but also the crystal growth [48]. Finally, the melting temperature (*T*_m_) is lower for the nanocomposites as compared to pure PEO, which points to a decrease of the lamellar thickness [49]. Overall, the differences between the different rGO-based nanocomposites are not very pronounced but GO induces more reduction of the degree of crystallinity as compared to the rGOs.

### 3.8. Effect of Polymer-Filler Interactions on the Glass Transition Temperature and the Dielectric Relaxation Strength of PEO-(r)GO Interfacial Polarization

**Figure 9:**
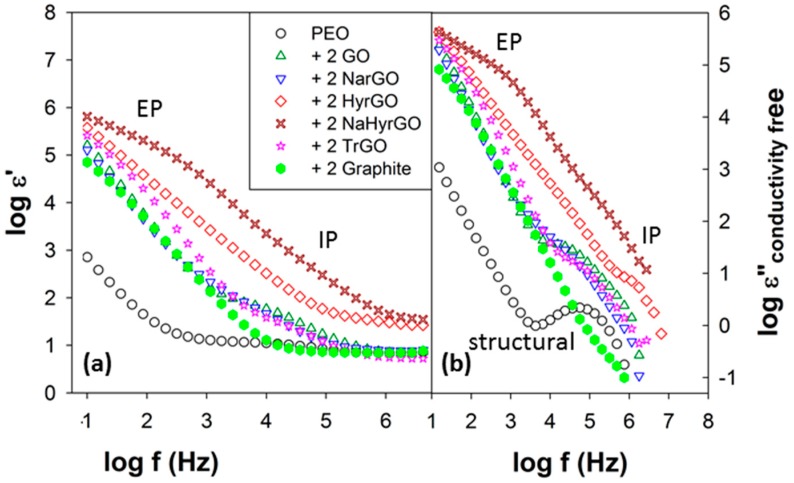
(**a**) Real part of dielectric permittivity and (**b**) Conductivity-free dielectric loss spectra calculated from (**a**) of PEO and PEO nanocomposites containing 2 wt % of various fillers at 100 °C. The electrode polarization (EP) at low frequencies, interfacial polarization (IP) in nanocomposites and structural relaxations in PEO at high frequencies have been demarcated in the permittivity responses.

In this section, the dielectric properties (AC dielectric permittivity ε’ and AC dielectric loss ε”) of the different nanocomposites will be discussed. The real and conductivity-free dielectric loss spectra of PEO and nanocomposites with 2 wt % of various fillers are shown in Figure 9a,b, respectively, at temperatures much higher (100 °C) than the *T*_g_ of PEO (≈−67 °C). The details of the various contributions of the ionic charges present in PEO and PEO-based nanocomposites to the permittivity spectrum are discussed in detail in literature [50,51]. At temperatures much higher than the *T*_g_ of the matrix PEO, an augmented mobility of the ionic charges present in PEO leads to their pronounced blocking at the electrode interfaces, resulting in contributions from electrode polarization at low frequencies in Figure 9. Since the measured dielectric properties in this region are not material properties, but are geometry-dependent, we will focus on the high-frequency response, which is intrinsic to the materials [26].

A Debye-like relaxation peak in PEO owing to segmental relaxations (associated with *T*_g_) is observed at ≈0.1 MHz. Interestingly, a relaxation peak at similar frequencies with a relaxation strength more than a decade higher than that of PEO is observed in all nanocomposites. The area under the relaxation peak (in Figure 9b) is an estimate of the number density of the contributing dipoles. Hence, in the nanocomposites this relaxation cannot be ascribed to any molecular or segmental relaxations of PEO, but only to the interfacial entrapment of the filler charges at the interface with the less conducting PEO matrix. Such an interfacial entrapment of charge carriers manifests itself when there is a strong mismatch in dielectric properties and conductivity of filler and matrix. Hence, two filler particles in close contact can be considered to be a microcapacitor with the filler particles forming the conductive electrodes encompassing the dielectric polymer [52]. A similar dielectric response was reported by Zhang et al. in polyvinyl alcohol (PVA)—multiwall carbon nanotubes (MWNTs) composites, in which the charges of the conductive fillers (MWNTs) were trapped at the PVA interface resulting in interfacial polarization of the conductive MWNTs charges [53]. Bharati et al. [52] were able to link the dielectric strength of the interfacial polarization to the connectivity of a MWNT microcapacitor network in compatibilized PMMA/PαMSAN blends with different connectivities of the MWNT-filled blend phase. The interfacial relaxation time increases exponentially with the gap between the filler particles, whereas the average time required for the double-layer formation of the PEO ionic charges at the electrodes is proportional to the ratio of sample thickness to the thickness of the double layer [27,52]. Due to the small length scales involved in interparticle microcapacitor formation, the interfacial relaxation occurs at much higher frequencies (short timescales) as compared to the electrode polarization, which allows to separate both in the frequency response spectrum.

The effect of polymer–filler interactions on the dielectric relaxation strength of the interfacial polarization of the more conductive filler in less conductive PEO is investigated here for 2 wt % nanocomposites. In Figure 9a, the step change of the dielectric permittivity at high frequencies gives an estimate of the dielectric relaxation strength. In Figure 9b, these relaxations show up as a relaxation peak, which is clearly visible after removal of the conductivity contribution to the dielectric loss. An accurate estimation of the dielectric strength has been obtained from the dielectric loss spectra (Figure 9b) using the Havriliak–Negami function [54]:

(4) ε′′=Im (Δε[1+(iωτHN)a]b)Δε=εs−ε∞

where Δε is the relaxation strength, ε_s_ and ε_∞_ are the dielectric constants at limiting low and high frequencies, respectively, *a* and *b* are the shape parameters for the asymmetry and breadth of the peak of the relaxation time distribution function, *τ*_HN_ is the relaxation time and *ω* is the angular frequency.

**Figure 10:**
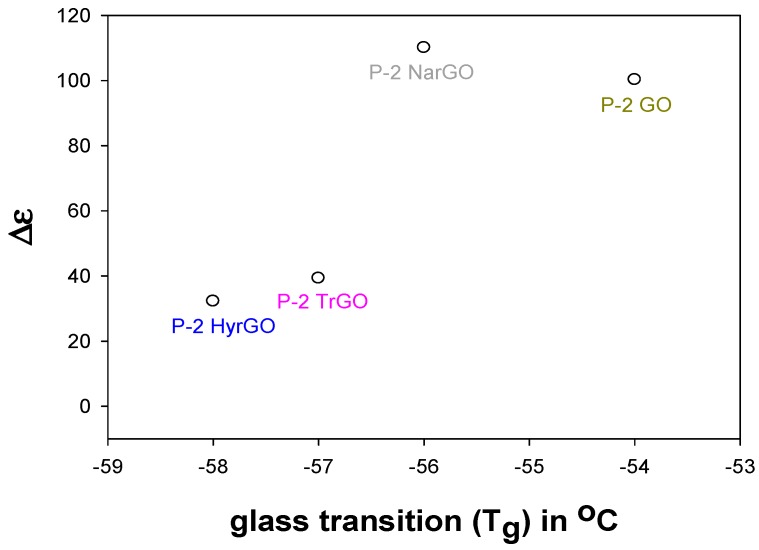
Dependence of dielectric relaxation strength of dielectric interfacial relaxation versus the calorimetric glass transition temperature (*T*_g_) for PEO composites with 2 wt % GO or rGO. The filler type is indicated with the data points.

Since it is well known that ionic conductivity in PEO is governed by the segmental mobility of the PEO chains [50], effects of polymer–filler interactions on the mobility of the PEO chains could affect the entrapment of charges at the filler–polymer interface and thus the interfacial dielectric relaxation. The segmental mobility of the polymer chains was quantified by means of the calorimetrically determined *T*_g_, an increase in *T*_g_ corresponds to a decrease in mobility of the polymer chains. To investigate the proposed correlation, the dielectric relaxation strength is plotted versus *T*_g_ in Figure 10 for various composites with 2 wt % fillers. We were unable to calculate the interfacial relaxation strength of graphite and NaHyrGO nanocomposites as electrode polarization masked the relatively slow interfacial relaxations. It can be seen in Figure 10 that a correlation exists between the dielectric relaxation strength and the calorimetric *T*_g_. A maximum dielectric strength and *T*_g_ is observed for NarGO and GO. This is due to the fact that these fillers are more polar (and hydrophilic) than TrGO and HyrGO, which results in favorable interactions with polar PEO. Graphite and NaHyrGO are expected to have a minimum amount of interactions with PEO, which agrees with the absence of a strong dielectric interfacial relaxation peak in these nanocomposites. Similarly, Putson et al. showed that a strong interaction between polyurethane (PU) and polyaniline (PANI) fillers results in a concomitant reduction of the available chain conformations (or mobility) of the PU chains adjacent to the PANI surface and an increase in the *T*_g_ of the overall composite. Interestingly, a concurrent increase in the dielectric constant of interfacial polarization occurred at the interface of PANI fillers with the PU matrix [55]. Thus, a slower mobility of the PU chains adjacent to the PANI surface allowed efficient entrapment of the charge carriers of PANI, resulting in an increased dielectric strength. This demonstrates that, apart from the well-known effects of filler concentration on the dielectric relaxation strength, improved interactions between filler and polymer could also contribute to enhanced dielectric properties since reduction of the polymer chain mobility can enhance entrapment of filler charges at the polymer–filler interface and thereby can increase the dielectric permittivity.

The effects of reduced chain mobility partially extend beyond *T*_g_ and dielectric relaxation strength to the crystallization behavior as the filler with the strongest interactions (GO) also has the largest effect on the degree of crystallinity of PEO (Table 1). However, these increased interactions do not transcend to a maximum increase in melt modulus. This can be attributed to the fact that the modulus is sensitive to polymer–polymer, polymer–filler and filler–filler interactions, whereas the relaxation strength is contingent on the polymer–filler interactions only. Furthermore, whereas *T*_g_ and dielectric strength of the filler–polymer–filler microcapacitors are governed by local phenomena, the viscoelastic moduli may be more dependent on the overall filler size, filler–polymer contact area and microstructure.

### 3.9. Effect of GO and Different rGOs on the Electrical Conductivity of PEO

**Figure 11:**
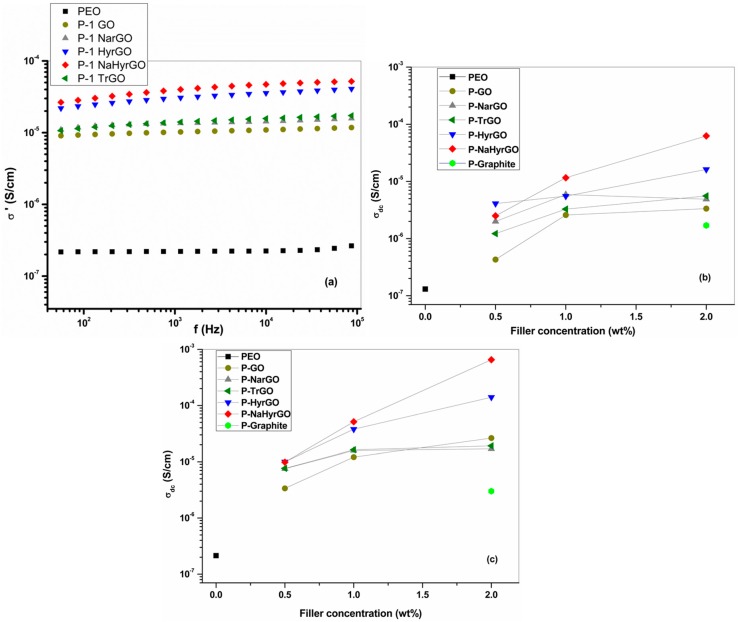
AC electrical conductivity (at 150 °C) of PEO-based nanocomposites with 1 wt % filler loading (**a**). (**b**,**c**) DC electrical conductivity (σ_dc_) versus filler concentration (wt %) for the nanocomposites at 100 and 150 °C.

Upon electrical percolation of the conductive filler, ensuring transport of charge carriers between filler particles via hopping or tunneling, electrically conductive polymer nanocomposites can be obtained [3]. The AC electrical conductivity at 150 °C for 1 wt % GO/rGO loaded PEO nanocomposites is shown in Figure 11a. All nanocomposites show a constant AC conductivity up to high frequencies whereas σ’ decreases at low frequencies due to electrode polarization, corresponding to the data in Figure 9 [51]. The GO and rGO fillers result in an increase of the electrical conductivity of more than two orders of magnitude. The order of the increase with the different fillers is qualitatively consistent with the electrical conductivity of the fillers in Figure 5, with the NaHyrGO resulting in the most conductive nanocomposite. However, whereas the fillers range in conductivity from 10^−4^ to 10^−1^ S/cm, the electrical conductivity of all nanocomposites in Figure 11 lies between 1 × 10^−4^ S/cm and 6 × 10^−4^ S/cm. This clearly shows that the contribution of the less conductive polymer in between the graphene sheets contributes significantly to the nanocomposite conductivity [56]. Furthermore, the less good dispersion of the most conductive filler (NaHyrGO, see Figure 6) may limit the maximum achievable electrical conductivity of the nanocomposites. Figure 11b,c show the electrical conductivity of GO and rGO loaded PEO nanocomposites as a function of filler loading. Similar to the storage modulus in Figure 6c, a power law dependency on the filler loading is not obtained for all fillers. This indicates the absence of a percolated fractal particle network for some of the nanocomposites. It can be seen that the use of NaHyrGO leads to a 2 wt % nanocomposite with a 40 times higher conductivity as compared to a PEO nanocomposite with graphite. The latter nanocomposite has the lowest conductivity due to the limited conductivity and unexfoliated nature of the graphite filler.

Figure 5 showed that in situ reduction at 150 °C leads to a substantial increase of the filler conductivity by heating. To investigate whether this is also reflected in the nanocomposite conductivity, Table 2 provides the effect of temperature on the filler as well as nanocomposite conductivity. It can be seen from the table that the electrical conductivity of GO drastically increased at 150 °C as compared to 100 °C, which is due to in situ thermal reduction of part of the different functional groups of GO. For other rGOs for which the functional groups were already reduced by different reducing agents, the increase in conductivity with increasing temperature (from 100 to 150 °C) is almost negligible. Table 2 shows that for the rGO-based nanocomposite, the temperature effect is much more pronounced for the nanocomposites as for the fillers. The decreased viscosity and resulting increased mobility of the rGO sheets can lead to the generation of more prominent conductive pathways in the nanocomposite by reorganization of the network [57]. Additionally, the electrons in graphene acquire more energy with increasing temperature, whereby their chance to surpass the potential energy barrier required for tunneling between different rGO sheets may increase [56,58]. These observations further demonstrate the dominant role of the less conductive polymer bridges between the nanoparticles in the overall nanocomposites conductivity.

## 4. Conclusions

A systematic investigation of the effect of GO and different rGOs on the electrical conductivity, linear viscoelastic properties and crystalline behavior of PEO was performed. Different functional groups of GO were selectively reduced by using thermal treatment at 1000 °C under inert atmosphere as well as by treating GO with different chemical reduction agents. Among the different reduction procedures, consecutive treatment with sodium borohydrate (NaBH_4_) and hydrazine monohydrate (N_2_H_4_·H_2_O) was the most efficient procedure. FT-IR and Raman spectroscopic analysis of different rGOs showed that a maximum amount of functional groups was removed and the conjugated graphitic structure, which was lost during oxidation, was effectively restored. As a consequence, the rGO obtained with this procedure exhibits better electrical conductivity (~0.1 S/cm at 150 °C) as compared to the other reduced GOs. Polymer nanocomposites based on PEO with different rGOs were prepared by solution casting. Melt linear viscoelastic moduli at low frequency increased due to polymer–particle and particle–particle interactions in the nanocomposites, indicating the presence of finely dispersed rGO sheets. PEO nanocomposites based on thermally reduced GO exhibited the largest modulus, indicating a better dispersion due to their more wrinkled and folded structure. In the solid state, the degree of crystallinity of PEO-based nanocomposites is reduced as compared to that of pure PEO, indicating reduced chain mobility of the PEO, which restricts crystallization. Correspondingly, the glass transition temperature was increased upon addition of the fillers. These effects were most pronounced for the polar fillers (GO and NarGO), which are expected to interact most strongly with PEO (via hydrogen bonds). In broadband dielectric spectroscopy, an interfacial relaxation originating from the entrapment of filler charges at the interface with less conductive PEO was obtained. Furthermore, a pronounced increase in the dielectric relaxation strength of this interfacial polarization was observed for composites with polar fillers having favorable interactions with the polar PEO matrix. Hence, a correlation occurred between the increase of the glass transition temperature and that of the dielectric strength of the interfacial polarization, thereby indicating that the strength of the interfacial entrapment of charges is related to the reduction of the mobility of the polymer chains. Finally, the electrical conductivity of the nanocomposites was characterized and increased by about two orders of magnitude due to the addition of the conductive fillers. The highest conductivity was obtained with the most conductive filler. However, filler type and temperature dependence of the electrical conductivity clearly showed the pronounced contribution of the entrapped polymer chains to the overall nanocomposite conductivity.

## Figures and Tables

**Table 1:** Thermal properties of PEO-based nanocomposites.

Samples	*T*_c_ (°C)	*T*_onset cryst._ (°C)	*T*_m_ (°C)	Δ*H*_m_ (J/g)	χ_c_ (%) (DSC)	χ_c_ (%) (XRD)
PEO	39.2	48.5	68.2	132.6	64.7	66.9
P-05 GO	47.3	49.9	64.8	106.6	52.0	55.0
P-05 NarGO	45.0	49.5	63.2	122.0	59.8	63.6
P-05 TrGO	46.8	50.4	66.2	106.6	52.2	58.7
P-05 HyrGO	41.2	45.5	63.0	132.3	64.8	71.0
P-05 NaHyrGO	46.0	50.0	62.8	119.8	58.7	65.0
P-2 GO	46.9	51.5	65.3	102.1	50.8	60.7
P-2NarGO	43.7	51.9	65.9	105.6	52.5	60.8
P-2 TrGO	48.0	52.2	66.7	103.5	51.5	60.7
P-2 HyrGO	44.6	49.4	62.4	127.5	63.5	65.8
P-2 NaHyrGO	44.3	50.2	61.3	111.6	55.5	63.1
P-2 Graphite	48.2	52.2	67.5	138.0	68.7	/

**Table 2:** Effect of temperature on the electrical conductivity of GO, rGOs and PEO-based nanocomposites.

Samples	σ’_150_/σ’_100_ (Fillers)	σ’_150_/σ’_100_ (PEO and Filled Composites)
PEO	-	1.6
P-2 GO	42.8	7.9
P-2 NarGO	1.2	3.4
P-2 HyrGO	1.5	8.6
P-2 NaHyrGO	1.4	10.5
P-2 TrGO	1.4	3.4
P-2 Graphite	15.7	1.7

## Supplementary Materials

The following are available online at www.mdpi.com/2073-4360/9/11/613/s1.
