# Supplementary material for: Effect of the GO Reduction Method on the Dielectric Properties, Electrical Conductivity and Crystalline Behavior of PEO/rGO Nanocomposites
Source: Polymers (Basel). 2017 Nov 14;9(11):613. doi: 10.3390/polym9110613 (PMC6418708; doi:10.3390/polym9110613)
Supplement: Supplementary file 1 [file polymers-09-00613-s001.pdf]

Effect of the GO reduction method on the dielectric properties, electrical conductivity and crystalline behavior of PEO/rGO nanocomposites

Asish Malas, Avanish Bharati, Olivier Verkinderen, Bart Goderis, Paula Moldenaers and Ruth Cardinaels

Supplementary information

XPS analysis: Experimental method

X ray photoelectron spectroscopy characterisation of the samples was carried out with a KAlpha device from Thermo Scientific using a monochromatic Al Kα X ray source (1486.6 eV) operating at 100 W and a hemispherical electron energy analyzer. High-resolution spectra of the C 1s and O 1s signals were recorded in 0.1 eV steps with a pass energy of 50 eV, while the takeoff angle was fixed at normal to the sample. Low energy electron flooding was used for charge compensation. After the linear baseline was subtracted, curve fitting was performed by a mixed Gaussian-Lorentzian product function. The energy scale was internally calibrated by referencing the binding energy of the C1s peak at 284.40 eV for contaminated carbon.

XPS analysis: Results

The high resolution C1s XPS spectra of graphite, GO and different chemically reduced GOs are presented in Figure S1. The C1s spectrum (Figure S1a) of graphite powder exhibits a main C1s peak at 284.4 eV implying that carbon is mostly present in the sp^2 hybridization state [1]. The broad C1s peak at ~285.5 eV can be attributed to carbon in C–O and C–O–C groups [2, 3]. The C1s XPS spectrum of GO (Figure S1b) distinctly indicates a significant degree of oxidation with six deconvoluted components that correspond to carbon atoms in various functional groups: non-oxygenated ring C (sp^3 C ~ 283.9 eV and sp^2 C ~ 284.6 eV) [4], C in C–O bonds (~285.5 eV), epoxide C (C-O-C, ~286.8 eV), carbonyl C (C=O, ~288.0 eV), and carboxylate C (O–C=O, ~289.0 eV) [5-7]. On the other hand, the high resolution C1s XPS spectrum of NarGO (Figure S1c), while showing the same oxygenated functional groups as GO, had smaller peak intensities for these fitting components, revealing partial de-oxygenation by the chemical reduction method. For HyrGO and NaHyrGO (Figure S1d-e), the high resolution C1s spectra show almost diminished peak intensities for these fitting components corresponding to the different functionalities which indicates a better reduction of the different functional groups of GO by hydrazine and dual reducing agents ($NaBH_4$ and hydrazine) as compared to only $NaBH_4$. For the high resolution C1s spectrum of GO and NaHyrGO, the determination of separate sp^3 C and sp^2 C contributions was possible. However, for NarGO and HyrGO, their combined involvement was denoted as C-C (sp^3 C & sp^2 C), since deconvolution into two separate peaks was not feasible. Similarly, for the high resolution C1s spectra of different reduced GOs, the C-O and C-O-C, and C=O, O-C=O and π-π^* (graphitic shake-up satellites, ~291 eV) contributions for NarGO; the C-O-C, C=O and O-C=O contributions for HyrGO; the C-O and C-O-C, and C=O, O-C=O and π-π^* contributions for NaHyrGO were not denoted separately, as the deconvolution into 2-3 peaks was also not possible [8, 9]. The (π-π^*) graphitic shake-up satellite contribution, noticed for different chemically reduced GOs around ~291 eV, evolves upon chemical reduction of GO. This implies that the delocalized π conjugation, a conventional trait of the aromatic carbon structure, was to some degree restored in various chemically reduced GOs [10-12]. Overall, the XPS spectra show a more pronounced presence of oxygen containing groups in NarGO whereas both HyrGO and NaHyrGO are quite similar in terms of their C1s spectra.

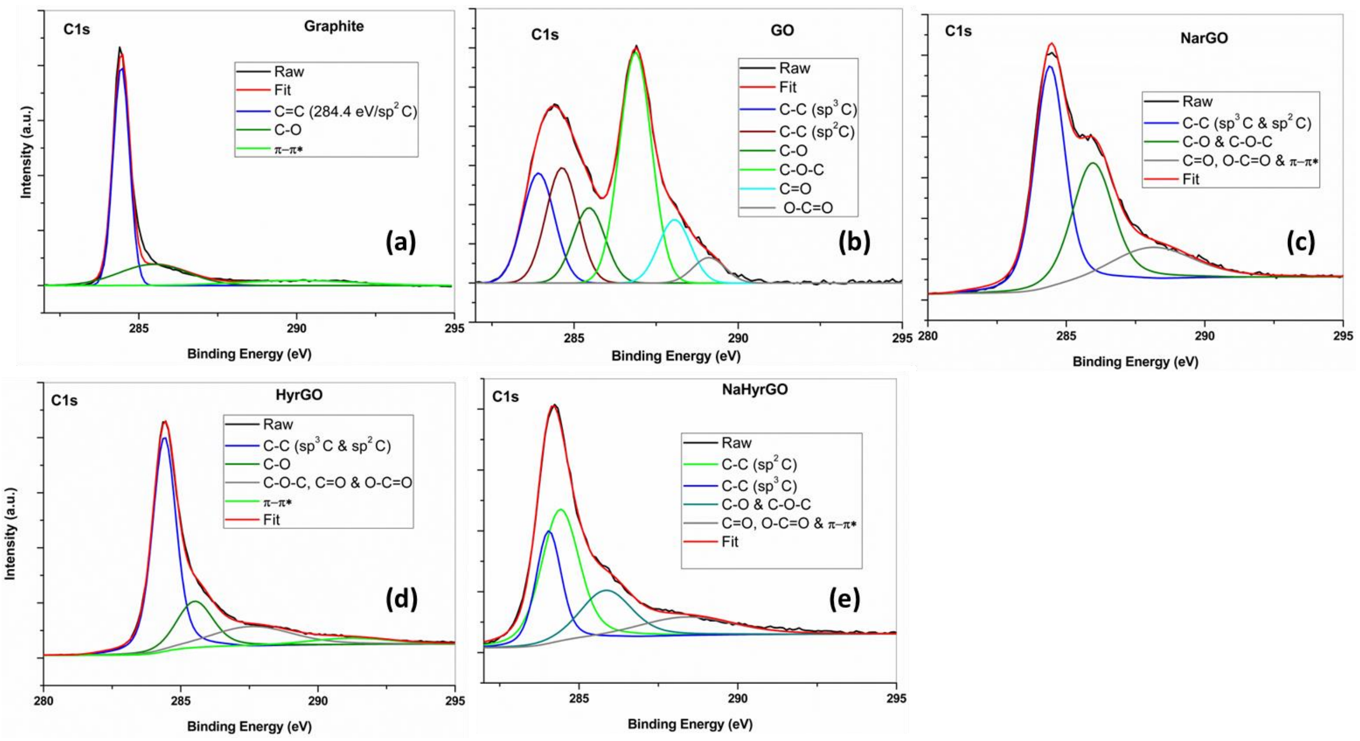

Figure S1: High resolution XPS (C1s) spectra of (a) graphite, (b) GO, (c) NarGO, (d) HyrGO and (e) NaHyrGO.

References

1. D. Bar-Tow, E. Peled, L. Burstein, A Study of Highly Oriented Pyrolytic Graphite as a Model for the Graphite Anode in Li-Ion Batteries, J. Electrochem. Soc., 146 (1999), p. 824.
2. H. Liu, Q. Xu, C. Yan, Y. Qiao, Corrosion behavior of a positive graphite electrode in vanadium redox flow battery, Electrochimica Acta, 56 (2011), p. 8783.
3. E. Desimoni, G.I. Casella, A. Monroe, A.M. Salvi, XPS determination of oxygen-containing functional groups on carbon-fibre surfaces and the cleaning of these surfaces, Surf. Interface Anal. 15 (1990) 627.
4. Fujimoto, A., Yamada, Y., Koinuma, M., & Sato, S. (2016). Origins of sp3C peaks in C1s X-ray Photoelectron Spectra of Carbon Materials. Analytical chemistry, 88(12), 6110-6114.
5. Chen, C. M. (2016). Structural Evolution of the Thermally Reduced Graphene Nanosheets During Annealing. In Surface Chemistry and Macroscopic Assembly of Graphene for Application in Energy Storage (pp. 51-71). Springer Berlin Heidelberg.
6. Huang, Y. L., Tien, H. W., Ma, C. C. M., Yang, S. Y., Wu, S. Y., Liu, H. Y., & Mai, Y. W. (2011). Effect of extended polymer chains on properties of transparent graphene nanosheets conductive film. Journal of Materials Chemistry, 21(45), 18236-18241.
7. Stankovich, S., Piner, R. D., Chen, X., Wu, N., Nguyen, S. T., & Ruoff, R. S. (2006). Stable aqueous dispersions of graphitic nanoplatelets via the reduction of exfoliated graphite oxide in the presence of poly (sodium 4-styrenesulfonate). Journal of Materials Chemistry, 16(2), 155-158.
8. Ganguly, A., Sharma, S., Papakonstantinou, P., & Hamilton, J. (2011). Probing the thermal deoxygenation of graphene oxide using high-resolution in situ X-ray-based spectroscopies. The Journal of Physical Chemistry C, 115(34), 17009-17019.
9. Tang, L., Li, X., Ji, R., Teng, K. S., Tai, G., Ye, J., ... & Lau, S. P. (2012). Bottom-up synthesis of large-scale graphene oxide nanosheets. Journal of Materials Chemistry, 22(12), 5676-5683.

10. Mattevi, C., Eda, G., Agnoli, S., Miller, S., Mkhoyan, K. A., Celik, O., ... & Chhowalla, M. (2009). Evolution of electrical, chemical, and structural properties of transparent and conducting chemically derived graphene thin films. Advanced Functional Materials, 19(16), 2577-2583.
11. Bagri, A., Mattevi, C., Acik, M., Chabal, Y. J., Chhowalla, M., & Shenoy, V. B. (2010). Structural evolution during the reduction of chemically derived graphene oxide. Nature chemistry, 2(7), 581-587.
12. Gao, W., Alemany, L. B., Ci, L., & Ajayan, P. M. (2009). New insights into the structure and reduction of graphite oxide. Nature chemistry, 1(5), 403-408.
